# Mesenchymal Stem Cell Membrane-Derived Composite System for Enhancing the Tumor Treatment Efficacy of Metal–Organic Framework Nanoparticles

**DOI:** 10.1049/nbt2/1069307

**Published:** 2024-12-05

**Authors:** Ying Tong, Meng Gao, Yingli Luo

**Affiliations:** ^1^Wuxi School of Medicine, Jiangnan University, Wuxi 214122, Jiangsu, China; ^2^State Key Laboratory of Food Science and Technology, Jiangnan University, Wuxi 214122, China; ^3^Department of Gastroenterology, The Second People's Hospital of Hefei, Hefei Hospital Affiliated to Anhui Medical University, Hefei 230011, Anhui, China

**Keywords:** cytotoxic effects, mesenchymal stem cell, metal-organic frameworks, tumor treatment

## Abstract

Mesenchymal stem cell (MSC) membrane-coated metal–organic frameworks (MOFs) represent an innovative approach to enhance the uptake and therapeutic efficacy of copper-based MOFs (Cu-MOFs) in tumor cells. By leveraging the natural homing abilities and biocompatibility of MSC membranes, Cu-MOFs can be effectively targeted to tumor sites, promoting increased cellular uptake. This coating not only facilitates superior internalization by cancer cells but also augments the therapeutic outcomes due to the enhanced delivery of copper ions. In vitro studies demonstrate that MSC membrane-coated Cu-MOFs (MSC-Cu-MOFs) significantly improve the cytotoxic effects on tumor cells compared to uncoated Cu-MOFs. This novel strategy presents a promising avenue for advancing the precision and effectiveness of cancer treatment modalities, showcasing potential for clinical applications in oncology.

## 1. Introduction

Cancer remains one of the most challenging health issues globally, with high morbidity and mortality rates despite advancements in medical science [1–3]. Traditional treatment modalities, including surgery, chemotherapy, and radiotherapy, have improved patient outcomes but often come with significant side effects and limitations, such as the development of resistance and nonspecific targeting of tumor cells. This has spurred the search for novel therapeutic strategies that can enhance the precision and efficacy of cancer treatments while minimizing adverse effects. Among the emerging approaches, nanotechnology-based therapies have garnered considerable attention, particularly the use of metal–organic frameworks (MOFs) due to their unique properties and versatility.

MOFs are crystalline materials composed of metal ions coordinated to organic ligands, forming porous structures. MOFs have been extensively explored for various biomedical applications, including drug delivery, imaging, and cancer therapy, due to their high surface area, tunable porosity, and functionalizable surfaces [4–7]. Copper-based MOFs (Cu-MOFs) are particularly promising in oncology because of the therapeutic properties of copper ions, which can induce oxidative stress and cytotoxicity in cancer cells [8–10]. However, the clinical translation of Cu-MOFs faces significant challenges, primarily related to their delivery and uptake by tumor cells. The efficacy of Cu-MOFs is often hampered by their poor targeting ability and rapid clearance by the immune system. To overcome these obstacles, innovative strategies to enhance the delivery and retention of Cu-MOFs in tumor tissues are essential.

Mesenchymal stem cells (MSCs) are multipotent stromal cells capable of differentiating into various cell types, including osteoblasts, chondrocytes, and adipocytes. MSCs possess unique properties that make them attractive for cancer therapy, such as their ability to home to tumor sites, modulate the immune system, and secrete therapeutic factors. These characteristics have prompted researchers to explore MSCs and their derivatives as potential delivery vehicles for cancer therapeutics [11–14]. The cell membrane of MSCs retains many of the parent cell's surface proteins and receptors, which can facilitate targeted delivery to specific tissues, including tumors [15–22]. By coating nanoparticles with MSC membranes, it is possible to exploit these targeting capabilities and improve the biodistribution and therapeutic efficacy of the encapsulated agents. The concept of using MSC membrane-coated Cu-MOFs (MSC-Cu-MOFs) represents a novel and promising approach to enhance the uptake and therapeutic efficacy of Cu-MOFs in cancer cells. The MSC membrane coating provides several advantages: (1) Targeting ability: MSC membranes exhibit natural homing abilities to tumor tissues, enabling the Cu-MOFs to accumulate more efficiently at the tumor site. This targeted delivery reduces the off-target effects and increases the local concentration of the therapeutic agent. (2) Biocompatibility: The biocompatible nature of MSC membranes can help evade the immune system, prolonging the circulation time of Cu-MOFs in the bloodstream and enhancing their delivery to tumor cells. (3) Enhanced uptake: The MSC membrane coating facilitates superior internalization by cancer cells. The presence of cell-adhesion molecules and other surface proteins on the MSC membranes can promote the endocytosis of Cu-MOFs by tumor cells, leading to higher intracellular concentrations of copper ions. (4) Improved therapeutic outcomes: By increasing the delivery and uptake of Cu-MOFs, the therapeutic efficacy is significantly enhanced. Cu ions released from the Cu-MOFs can induce oxidative stress and apoptosis in cancer cells, improving the overall cytotoxic effects.

In this work, we have partly demonstrated the potential of MSC-Cu-MOFs in enhancing cancer therapy. In vitro experiments have shown that these coated nanoparticles exhibit significantly improved cytotoxic effects on various cancer cell lines compared to uncoated Cu-MOFs. The enhanced uptake and retention of Cu-MOFs within cancer cells lead to increased oxidative stress and apoptosis, thereby, reducing tumor cell viability. While the use of MSC-Cu-MOFs in cancer therapy is promising, several challenges remain. The scalability and reproducibility of the coating process need to be optimized to ensure consistent production of the nanoparticles. Additionally, long-term safety and efficacy studies are necessary to evaluate the potential for clinical use. The integration of MSC membranes with Cu-MOFs represents a significant advancement in the field of nanomedicine for cancer therapy. This innovative approach leverages the natural targeting abilities and biocompatibility of MSC membranes to enhance the delivery and efficacy of Cu-MOFs. The promising results provide a strong foundation for further development and potential clinical translation, offering new hope for more effective and less toxic cancer treatments.

## 2. Materials and Methods

### 2.1. Preparation of Cu-MOF and MSC-Cu-MOF

Cu-MOFs were synthesized using a solvothermal method. Briefly, copper nitrate trihydrate (Cu (NO_3_)_2_·3H_2_O) and 2-methylimidazole (2-MIM) were dissolved in a mixture of ethanol and water. The solution was then transferred to a teflon-lined autoclave and heated at 120°C for 24 h. The resultant Cu-MOF crystals were collected by centrifugation, washed with ethanol and water several times to remove any unreacted precursors, and then dried under vacuum at 60°C.

MSCs were cultured to confluence and then harvested. The cells were lysed using a hypotonic buffer and subjected to ultracentrifugation to isolate the cell membranes. The isolated membranes were washed and resuspended in phosphate-buffered saline (PBS). The extracted MSC membranes were mixed with the presynthesized Cu-MOFs in a solution of PBS. The mixture was subjected to sonication and extrusion through a polycarbonate membrane using a liposome extruder to ensure uniform coating of the Cu-MOFs with MSC membranes. The resulting MSC-Cu-MOF nanoparticles were collected by centrifugation and washed to remove excess membranes.

### 2.2. Characterization of Cu-MOF and MSC-Cu-MOF

The hydrodynamic diameter and zeta potential of the Cu-MOF and MSC-Cu-MOF nanoparticles were measured using dynamic light scattering (DLS). Samples were dispersed in deionized water and analyzed using a Zetasizer Nano ZS (Malvern Instruments). The average particle size and polydispersity index (PDI) were recorded, and the zeta potential was determined to assess the surface charge and stability of the nanoparticles.

The morphology and structure of the Cu-MOF and MSC-Cu-MOF nanoparticles were examined using transmission electron microscopy (TEM). A drop of the nanoparticle suspension was placed onto a carbon-coated copper grid and allowed to dry. The samples were then imaged using a JEOL JEM-2100F TEM at an accelerating voltage of 200 kV. The images were analyzed to determine the shape and size distribution of the nanoparticles. This comprehensive methodology ensures the successful synthesis and characterization of Cu-MOF and MSC-Cu-MOF nanoparticles, providing a basis for further investigation into their biomedical applications.

### 2.3. Cellular Uptake of Cu-MOF and MSC-Cu-MOF

To assess the differential uptake of rhodamine-loaded Cu-MOFs (RhoB-Cu-MOFs) and MSC membrane-coated Cu-MOFs by melanoma cells, we utilized flow cytometry. The experimental protocol was as follows: RhoB-Cu-MOFs and MSC membrane-coated Rhob-Cu-MOFs (MSC-Rhob-Cu-MOFs) were synthesized and characterized for their size, zeta potential, and fluorescence intensity.

B16–F10 melanoma cells were cultured in Dulbecco's modified Eagle medium (DMEM) supplemented with 10% fetal bovine serum (FBS) and 1% penicillin–streptomycin at 37°C in a humidified atmosphere containing 5% CO_2_.

Melanoma cells were seeded into 24-well plates at a density of 1 × 10^4^ cells per well and allowed to adhere overnight. The cells were then incubated with RhoB-Cu-MOFs and MSC-RhoB-Cu-MOFs at a concentration of 80 µg/mL for 6 h at 37°C. After incubation, the cells were washed three times with cold PBS to remove free nanoparticles. The cells were then detached using trypsin–EDTA, collected, and resuspended in PBS containing 2% FBS.

The cell suspensions were analyzed using a flow cytometer (BD FACSCanto II). Rhodamine fluorescence was detected using the appropriate excitation and emission wavelengths (excitation: 540 nm and emission: 625 nm). A total of 10,000 events were recorded for each sample. The mean fluorescence intensity (MFI) of rhodamine in melanoma cells treated with RhoB-Cu-MOFs and MSC-RhoB-Cu-MOFs was compared. Flow cytometry data were analyzed using FlowJo software to quantify the uptake efficiency.

To further evaluate the differential uptake of RhoB-Cu-MOFs and MSC-RhoB-Cu-MOFs by melanoma cells using laser scanning confocal microscopy, melanoma cells were seeded onto glass-bottomed culture dishes at a density of 1 × 10^5^ cells per dish and allowed to adhere overnight. Then, the cells were incubated with RhoB-Cu-MOFs and MSC-RhoB-Cu-MOFs at a concentration of 50 µg/mL for 8 h at 37°C. After incubation, the cells were washed three times with cold PBS to remove any unbound nanoparticles. The cells were then fixed with 4% paraformaldehyde for 15 min at room temperature. The cell membranes were stained with a fluorescent membrane dye to visualize the cell boundaries. The nuclei were counterstained with DAPI. The samples were imaged using a laser scanning confocal microscope. The fluorescence intensity and distribution of rhodamine within the cells were analyzed using NIS Viewer software. Colocalization analysis was performed to determine the extent of nanoparticle uptake and intracellular localization.

### 2.4. Cell Viability

Melanoma cells were seeded into 24-well plates at a density of 1 × 10^5^ cells per well and allowed to adhere overnight. The cells were treated with RhoB-Cu-MOFs and MSC-RhoB-Cu-MOFs at a concentration of 100 µg/mL for 12 h. After treatment, the cells were washed with cold PBS and incubated with 1 µg/mL PI solution for 15 min at room temperature in the dark. PI intercalates with DNA in dead cells, emitting fluorescence upon excitation. The stained cells were observed under a fluorescence microscope. PI fluorescence was excited at 535 nm and emitted at 617 nm. Images were captured for further analysis.

Moreover, the cells were treated with RhoB-Cu-MOFs and MSC-RhoB-Cu-MOFs at a concentration of 100 µg/mL for 24 h. Following treatment, the cells were harvested using trypsin–EDTA and resuspended in fresh medium. An equal volume of 0.4% trypan blue dye was added to the cell suspension and mixed gently. The mixture was incubated for 2–3 min at room temperature, and then 10 µL of the stained cell suspension was loaded onto a hemocytometer.

## 3. Results and Discussion

**Figure 1:**
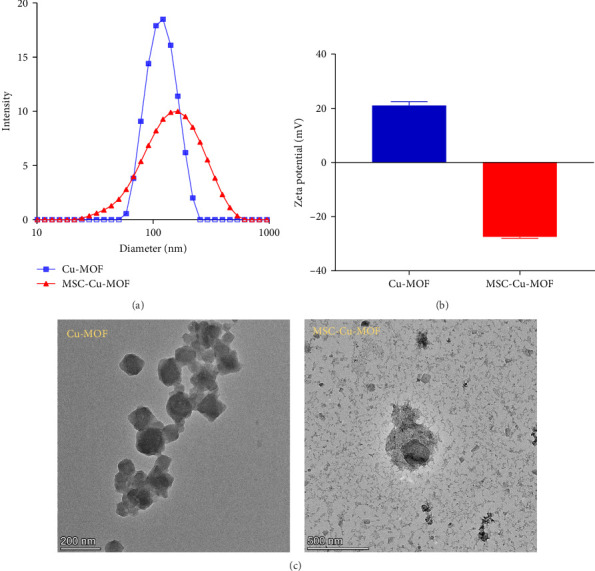
Characterization of Cu-MOF and MSC-Cu-MOF nanoparticles. (A) the average size of Cu-MOF and MSC-Cu-MOF nanoparticles detected by dynamic light scatting. (B) Zeta potential measurement of Cu-MOF nanoparticles and MSC-Cu-MOF nanoparticles. (C) TEM image of synthesized Cu-MOF nanoparticles and MSC-Cu-MOF nanoparticles. Cu-MOF, copper-based metal–organic framework; MSC, mesenchymal stem cell; MSC-Cu-MOF, MSC membrane-coated Cu-MOF; TEM, transmission electron microscopy.

The average size of the Cu-MOF particles was determined to be 122 nm. Upon coating with MSC membranes, the size increased to 164 nm, indicating the successful encapsulation of the nanoparticles within the cellular membrane (Figure 1A). This size increase is consistent with the addition of the membrane layer around the Cu-MOF core.

In addition to the size measurements, the zeta potential analysis provided further evidence of the successful coating process. The uncoated Cu-MOF nanoparticles exhibited a positive zeta potential of +20.95 mV, which is characteristic of the surface charge of the MOF. After coating with the MSC membrane, the zeta potential shifted to a negative value of −27.45 mV (Figure 1B). This significant change in surface charge indicates the presence of the negatively charged cellular membrane, confirming the successful membrane coating.

TEM images further corroborated these findings, revealing the uniform spherical morphology of the Cu-MOF nanoparticles and the distinct membrane-like structure on the surface of the MSC-coated nanoparticles (Figure 1C). The membrane coating not only increased the particle size but also imparted a smooth and continuous layer around the core nanoparticles, providing additional stability and biocompatibility.

Overall, the successful synthesis and characterization of MSC-Cu-MOF nanoparticles were demonstrated by the increase in particle size, the shift in zeta potential, and the TEM observations. These modifications are expected to enhance the targeting efficiency and therapeutic efficacy of the nanoparticles in tumor treatment.

**Figure 2:**
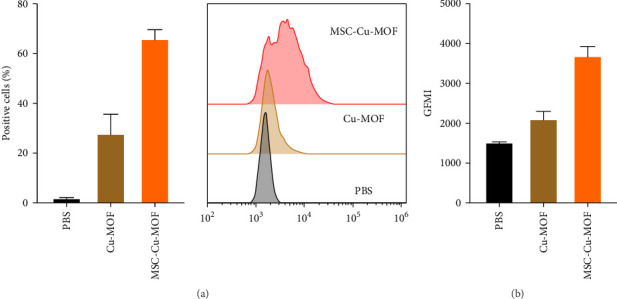
Flow cytometry detection of Cu-MOF and MSC-Cu-MOF uptake by melanoma cells. (A) Flow cytometry analysis of rhodamine-positive cells after treatment with Cu-MOF and MSC-Cu-MOF. (B) Quantification of rhodamine-positive cells. Cu-MOF, copper-based metal–organic framework; MSC-Cu-MOF, mesenchymal stem cell membrane-coated Cu-MOF; PBS, phosphate-buffered saline.

Flow cytometry analysis revealed a significant difference in the uptake of Cu-MOF and MSC-Cu-MOF by melanoma cells. The results indicated that melanoma cells exhibited a higher propensity for internalizing MSC-Cu-MOF compared to uncoated Cu-MOF. Specifically, the proportion of rhodamine-positive cells was markedly higher in the MSC-Cu-MOF group, reaching over 60%, which is nearly twice as high as the Cu-MOF group (Figure 2A). This substantial increase in cellular uptake demonstrates the effectiveness of the MSC membrane coating in enhancing the internalization of the nanoparticles by tumor cells.

In addition to flow cytometry, fluorescence quantification further confirmed the enhanced uptake of MSC-Cu-MOF by melanoma cells. The fluorescence intensity within the tumor cells was significantly higher in the MSC-Cu-MOF group compared to the Cu-MOF group (Figure 2B). These findings suggest that the MSC membrane coating not only facilitates superior internalization but also potentially enhances the therapeutic efficacy of Cu-MOF by increasing the intracellular concentration of the nanoparticles.

**Figure 3:**
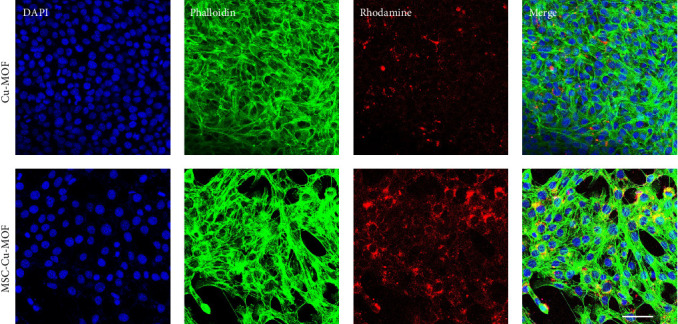
Confocal laser scanning microscopy images of melanoma cells incubated with different nanoparticles.

Furthermore, confocal laser scanning microscopy was used to assess the uptake of different nanoparticles by melanoma cells. The results revealed that melanoma cells incubated with MSC-Cu-MOF exhibited significantly higher intracellular fluorescence compared to those treated with uncoated Cu-MOF. The enhanced fluorescence indicates a greater accumulation of MSC-Cu-MOF within the tumor cells. Specifically, the rhodamine fluorescence intensity was markedly increased in the MSC-Cu-MOF group, suggesting that the MSC membrane coating significantly improves the nanoparticle uptake and retention in melanoma cells (Figure 3). This enhanced accumulation is likely due to the biomimetic properties of the MSC membrane, which facilitates better interaction and internalization by the tumor cells, thereby, enhancing the therapeutic potential of the nanoparticles.

**Figure 4:**
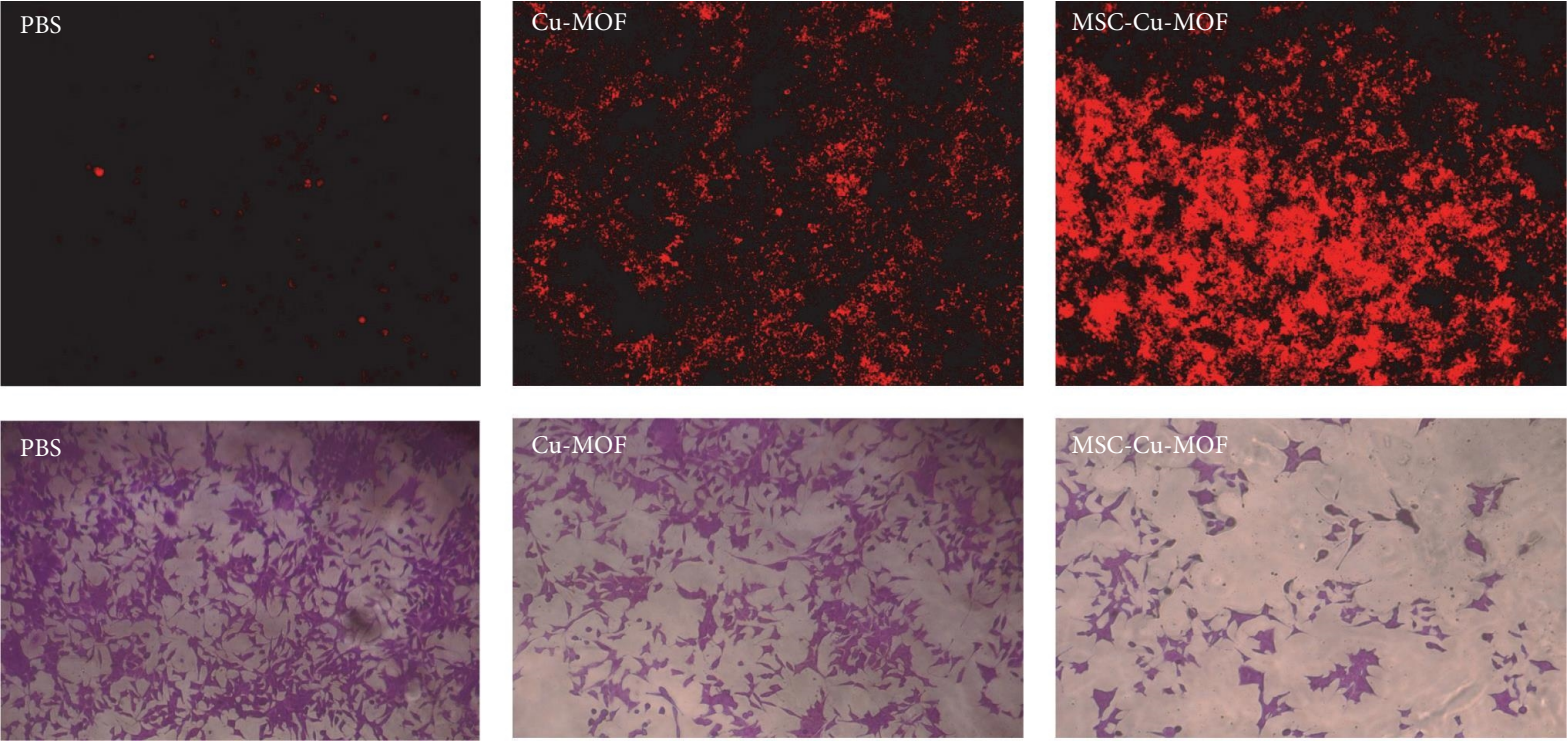
Analysis of PI/trypan-stained melanoma cells treated with different nanoparticles.

The efficacy of MSC-Cu-MOF in inducing melanoma cell death was further assessed using both PI staining and trypan blue exclusion assays. Confocal analysis of PI-stained cells revealed that MSC-Cu-MOF treatment resulted in a significantly higher proportion of dead cells compared to uncoated Cu-MOF. Specifically, the percentage of PI-positive cells was substantially greater in the MSC-Cu-MOF group, indicating enhanced cytotoxicity. In parallel, trypan blue staining further confirmed these findings, with the MSC-Cu-MOF-treated group exhibiting the lowest number of viable cells (Figure 4). These results suggest that the MSC membrane coating not only improves nanoparticle uptake by melanoma cells, but also significantly enhances their ability to induce cell death, highlighting the potential of MSC-Cu-MOF as a more effective therapeutic agent for melanoma treatment.

## 4. Conclusion

In conclusion, the use of MSC membrane-coated MOFs presents a groundbreaking strategy to enhance the therapeutic efficacy of Cu-MOFs in cancer treatment. By harnessing the homing abilities and biocompatibility of MSC membranes, this approach significantly improves the targeted delivery and uptake of Cu-MOFs by tumor cells. The superior internalization and enhanced delivery of copper ions result in markedly increased cytotoxic effects on cancer cells, as demonstrated in vitro. This innovative method not only amplifies the therapeutic outcomes but also holds great promise for clinical applications, offering a more precise and effective modality for oncology. Future studies and clinical trials are warranted to further validate the potential of MSC-Cu-MOFs and pave the way for their integration into standard cancer treatment protocols.

## Data Availability

The data that support the findings of this study are available on request from the corresponding author. The data are not publicly available due to privacy or ethical restrictions.
